# Hyperreactio luteinalis with preeclampsia

**DOI:** 10.4103/0974-2700.66545

**Published:** 2010

**Authors:** Alev Atis, Filiz Cifci, Yavuz Aydin, Gulseli Ozdemir, Nimet Goker

**Affiliations:** Obstetrician and Gynecologist, Sisli Etfal Training and Research Hospital, Istanbul, Turkey; 1 Obstetrician and Gynecologist, Istanbul University, Istanbul, Turkey

**Keywords:** Hyperreactio luteinalis, preeclampsia, pregnancy

## Abstract

Hyperreactio luteinalis (HL) is a condition associated with bilateral and, in rare cases, unilateral ovarian enlargement due to theca lutein cysts. HL is a benign condition, usually found incidentally at cesarean section, which can appear anaplastic and lead to unnecessary ovarian resection. A 24-year-old woman with 35 weeks of gestation attended with bilaterally enlarged ovaries. She had preeclampsia and preterm contractions. Due to breech presentation of baby and nulliparity and possible severe preeclampsia, she delivered by cesarean section. The ovaries had an anaplastic appearance and a biopsy was taken during cesarean section. Pathology revealed multiple benign theca lutein cysts. There are 51 reported cases of HL associated with a normal pregnancy in the literature. It is estimated that approximately 60% of the cases of HL is not associated with trophoblastic disease and occurs with normal singleton pregnancy. Only three of them were found to be associated with preeclampsia and this is the fourth case. HL may help explain the underlying cause of preeclampsia in these cases. There are multiple benign ovarian lesions in HL, which can mimic ovarian neoplasms. Accordingly, it is important to exclude these from the differential diagnosis via a wedge biopsy and frozen section to avoid unnecessary surgical excision.

## INTRODUCTION

Hyperreactio luteinalis (HL) refers to moderate to marked cystic enlargement of the ovaries due to multiple benign theca lutein cysts and is most often associated with hydatidiform mole or choriocarcinoma. The cause of this condition is unknown, but is believed to be related to elevated levels of, or abnormal ovarian response to, human chorionic gonadotropin (hCG) and pituitary gonadotropins. The ovaries in HL are symmetrical with uniform sized theca lutein cysts.[1]

Most of the pregnant women with HL are asymptomatic, and enlarged ovaries with multiple cysts are usually accidentally discovered on routine ultrasound (US) examination. This benign condition may imitate enlarged ovaries in ovarian hyperstimulation syndrome (OHSS) or ovarian malignancy. OHSS is usually a consequence of iatrogenic ovarian induction, but in rare occasions it can occur spontaneously, particularly in hypothyroidism, pituitary gonadotropin secreting adenoma, pregnant women with polycystic ovarian syndrome (PCOS), or mutation in follicle situmulating hormone (FSH) or luteinizing hormone receptors.[2–4] We report here on an HL case admitted to the emergency clinic and discuss the management.

## CASE REPORT

A 24-year-old woman G1P0, with 35 weeks of pregnancy, was referred to our hospital’s emergency clinic for bilateral inguinal pain which was more severe on the left. Obstetrics consultant found rebound on left inguinal region on physical examination and bilaterally enlarged ovaries with multiple cysts by ultrasonography. The right ovary was 160 × 130 mm and the left ovary was 140 × 110 mm in size. Breech presenting fetus with an estimated weight of 2500 g and normal amount of amniotic fluid were found. Minimal peritoneal fluid was present in the Pouch of Douglas. Cervical dilatation was not found by vaginal examintion. She had (++) pretibial edema and (++) proteinuria was deteacted in urine analysis. Her medical history in the first trimester was associted with hyperemezis gravidarum needing hospital care. She had an appendectomy a year ago and therapy for PCOS with oral contraceptives before pregnancy. She had no signs of thyroid disorder. She had signs of hyperandrogenism on face. On admission, her vital signs were stable, blood pressure was 150/100 mm Hg, heart rate was 88 beats/minute, uterine contractions were 12 minutes apart with baseline level reaching 30 mm Hg on nonstress test. Laboratory data showed normal liver and renal function and normal coagulation parameters with a hemoglobin level of 10 g/dl, platelet level of 227,000, white blood cell count of 9500, and (++) proteinuria on urine analysis. In the light of these findings, our team decided to perform cesarean section due to nulliparous breech presentation and possible severe preeclampsia. A male baby, weighting 2620 g, with an Apgar score of 7 at 1 minute and 9 at 5 minutes was delivered. Placental histology showed focal villous infarction and ischemic changes without infection or trophoblastic disease, and the histologic analysis of a biopsy sample from the ovarian tumor confirmed hypertrophic luteinized cells. Her blood pressure and proteinuria did not get any worse postpartum, ranging between 130/90 and 140/90 mm Hg levels 2 days postpartum. Her blood pressure recovered to 120/80–110/70 mm Hg on third and fourth postpartum days. Her urinary protein level of 24 hours was found to be 900 mg/l, and she was diagnosed to be having mild preeclampsia postpartum. It took approximately 3 months for her enlarged ovaries to return to normal size.

## DISCUSSION

HL is most often bilateral and found incidentally at the time of cesarean section. However, HL may present during any trimester as an abdominal mass or acute abdomen. The natural course is postpartum regression. Recognition of HL is important, since misinterpretation at laparotomy or erroneous histologic diagnoses has resulted in unnecessary surgery, often with sterilization of most of the cases. A conservative approach is indicated with wedge biopsy and frozen section diagnosis. Oophorectomy is necessary only to remove infarcted tissue or to control hemorrhage.

This condition has been demonstrated to develop with hypothyroidism, PCOS, FSH secreting adenoma, or mutation in the FSH receptor.[4] Our patient had no history of induction of ovulation or and showed no hypothyroidism or increase in FSH levels but she had therapy for PCOS with oral contraceptives before pregnancy.

In rare cases, HL could be associated with hyperthyroidism, hyperandrogenism and hirsutism, IUGR, eclampsia, or hemolysis, elevated liver enzymes, and low platelets (HELLP) syndrome.[3,5–7] The patient was symptomatic with hyperemesis gravidarum in the first trimester and developed signs of hyperandrogenemia and hirsutism and preeclampsia.

There are 51 reported cases of HL associated with a normal pregnancy, in the literature. It is estimated that approximately 60% of the cases of HL unassociated with trophoblastic disease occurs with normal singleton pregnancy. There are multiple benign ovarian lesions including HL, which can mimic ovarian neoplasms. Accordingly, it is important to exclude these through differential diagnosis via a wedge biopsy and frozen section to avoid unnecessary surgical excision. HL is most often bilateral and found incidentally at the time of cesarean section. However, HL may present during any trimester as an abdominal mass or acute abdomen. The natural course is postpartum regression.

Using MEDLINE, we found that only three cases of HL with PE or related disease have been reported in the literature.[3,5–7] The first case of HL with PE, found in a second trimester pregnancy, occurred in a woman with inherited thrombophilia, who developed severe, early onset PE; the clinical features of PE improved after administration of low-molecular-weight heparin. The second case had been diagnosed during the second trimester of pregnancy, the woman developed eclampsia after mild preeclampsia, at a gestational age of 28 weeks. The third case had HL with high hCG levels in the first trimester and developed HELLP syndrome after severe preeclampsia associated with intrauterine growth retardation at 34 weeks gestation, and the fourth case had been presented with IUGR.[3,5–7]

The elevation of hCG derived from placenta in a normal pregnancy could be caused by poor placentation, which could lead to development of preeclampsia. Observation of HCG levels during the second trimester has been shown to be useful in identifying pregnant women at risk of developing PE.[2]

Masuyama *et al*., observed an imbalance of angiogenic factors and hCG levels that were 10 times higher than normal during the first trimester, and the high hCG levels continued until term, along with bilateral enlarged ovaries. Their case was the first observation of HL associated with elevated hCG level and a severe imbalance of angiogenic factors. This case developed into early onset, severe PE. Therefore, too much elevated hCG level might be a risk or predictive factor for PE.[8]

It is estimated that approximately 60% of the cases of HL is not associated with trophoblastic disease, and these cases presented with normal singleton pregnancy. Only three of them were associated with preeclampsia and our case is the fourth one. HL may help to explain the underlying cause of preeclampsia. Our case is important for representing the symptoms of hyperemezis, preeclampsia and preterm contractions, all of which seem to be associated with high HCG levels and may be associated with incomplete placental invasion and inadequate angiogenesis.
